# Impact on the Intervertebral Foramen After Posterior Cervical Fixation Surgery Using Cervical Pedicle Screws

**DOI:** 10.7759/cureus.89536

**Published:** 2025-08-07

**Authors:** Yuki Kono, Terumasa Ikeda, Katsuhiro Ichikawa, Hiroshi Miyamoto

**Affiliations:** 1 Radiology Center, Kindai University Hospital, Osakasayama, JPN; 2 Orthopedic Surgery, Kindai University Hospital, Osakasayama, JPN; 3 Quantum Medical Technology, Institute of Medical, Pharmaceutical and Health Sciences, Kanazawa University, Kanazawa, JPN; 4 Orthopaedic Surgery, Kobe Rosai Hospital, Kobe, JPN

**Keywords:** c5 nerve root palsy, cervical pedicle screws, intervertebral foraminal stenosis, posterior cervical fixation, three-dimensional computed tomography analysis

## Abstract

Purpose

We aimed to compare postoperative changes in intervertebral foraminal areas and the mechanisms of foraminal stenosis following fixation surgery for cervical spondylotic myelopathy (CSM) with local kyphosis and instability, using three-dimensional computed tomography (3DCT).

Methods

We retrospectively analyzed 55 patients who underwent posterior cervical spinal fixation using pedicle screws. A total of 71 spinal levels (C2/3 to C7/T1) and 144 intervertebral foramina with anchor screws inserted in the upper and lower vertebrae were examined. The C2/7 angle and segmental local angle (SA) were measured, and the change in SA (postoperative minus preoperative, defined as the α angle) was calculated. 3DCT was used to measure the craniocaudal (CC) and anteroposterior (AP) diameters and the area of the intervertebral foramina pre- and postoperatively. The percentage change in area was calculated as the difference between postoperative and preoperative areas, divided by the preoperative area. Correlations between the α angle and percentage change in foraminal area were analyzed.

Results

The primary mechanism of foraminal stenosis was a “lag effect” in which the upper fixed vertebra was pulled backward due to posterior slippage. The foraminal area tended to increase when the α angle was corrected to less than 0°. However, large corrections with an α angle exceeding 10° significantly reduced the foraminal area compared to corrections to less than 0° (p < 0.01). The greatest risk of foraminal narrowing was observed when the α angle was ≥ 10°, resulting in a 16.4% (5.4 mm^2^) reduction in foraminal area. This phenomenon was also observed at the C4/5 level, where the intervertebral foraminal area decreased by 26.9% (8.6 mm^2^) and the α angle was approximately 10° in patients with C5 palsy.

Conclusion

These findings enhance our understanding of foraminal area changes after posterior fusion using cervical pedicle screws and may help prevent postoperative nerve root palsy, particularly C5 palsy.

## Introduction

Cervical pedicle screws (CPS) provide strong stabilization and are effective in treating cervical spondylosis with instability or for spinal reconstruction [1]. Cervical spondylotic myelopathy (CSM) with local kyphosis is associated with poor surgical outcomes. Compared to laminoplasty (LP) alone, posterior corrective fixation using CPS yields superior clinical outcomes in such cases [2,3]. However, postoperative nerve root palsy remains a troublesome complication following posterior decompression and fixation [4-8]. C5 nerve palsy is the most common, followed by C6, C7, and C8 palsies [7-10]. The pathogenesis of C5 palsy after LP is believed to involve posterior spinal cord shift and nerve root traction [11,12], occurring in 4-10% of LP cases, mostly transient [13-16]. In contrast, the incidence of C5 palsy is higher (up to ~12%) following posterior fixation for CSM with instability [13-16]. CPS-based posterior reconstruction is often preferred for correcting cervical kyphosis due to its biomechanically stronger fixation compared to lateral mass screws [17,18]. However, this stronger correction force can result in iatrogenic foraminal stenosis, especially when overcorrecting local kyphosis [5]. Excessive correction is caused by the strong corrective force of the CPS, making it difficult to optimize the corrective force and correction angle. Unlike LP, the mechanism of C5 palsy in fixation surgery likely involves structural changes in the foraminal area due to these strong corrective forces, which are difficult to modulate. Despite the clinical significance, detailed data on foraminal area changes following CPS fixation surgery in patients with kyphosis or instability are lacking. Traditional two-dimensional computed tomography (2DCT) is insufficient for accurate evaluation in patients with cervical deformities. Therefore, three-dimensional computed tomography (3DCT), which allows precise visualization and consistent measurement of the intervertebral foramen, is essential. In this study, we aimed to investigate postoperative changes in intervertebral foraminal morphology using 3DCT to better understand and potentially prevent C5 nerve palsy following CPS fixation in patients with CSM accompanied by kyphosis (including severe deformities) or instability.

## Materials and methods

Patient demographics and eligibility criteria

In this retrospective study, we included 55 patients (26 men and 29 women; mean age, 72.3 years; range, 54-87 years) who underwent cervical fixation at our hospital between 2013 and 2020. All patients underwent decompression and posterior spinal fixation using CPS, with an average follow-up period of 5.2 years (range 24‒140 months). Patients who underwent posterior fixation extending from the occiput to C2 and C1/2 were excluded. Patients undergoing lateral mass screw fixation, combined anterior fusion, and cervical infectious cases were also excluded. Indications for surgery included CSM with instability (n = 24), CSM with local kyphosis (n = 16), destructive spondyloarthropathy (n = 4), and dropped head syndrome (n = 11). The study protocol was approved by the institutional review board, and informed consent was obtained from all patients.

Surgical procedures

All patients underwent posterior spinal fixation using a pedicle screw-rod system. A total of 400 CPSs were used (7.2 screws/patient). Posterior laminoplasty was also performed in all cases. Fixation levels ranged from C2/3 to C7/T1 with the CPS. Cases involving lateral mass screw or combined anterior fusion were excluded. This study focused solely on patients treated with posterior fusion using a CPS rod system. Cervical fixation was performed from C2 to T8, although there were some levels where screws were not inserted. Ultimately, 144 intervertebral joints (C2/3 to C7/T1) were directly fixed with pedicle screws (Table 1).

**Table 1:** Demographic data concerning the cervical fixation area for 55 patients We analyzed 55 patients who underwent posterior cervical spinal fixation using pedicle screws. In total, 71 spinal levels (C2/3 to C7/T1) and 144 intervertebral foramina with anchor screws inserted in the upper and lower vertebrae were examined. C: cervical; T: thoracic; n: number.

area	n	area	n	area	n
C2–4	2	C3–4	4	C4–5	6
C2–5	3	C3–5	5	C4–T7	1
C2–6	2	C3–6	10		
C2–7	4	C3–7	5		
C2–T1	9	C3–T1	2		
		C3–T5	2		

Sample size justification

This study was exploratory and did not involve formal sample size calculations. All patients who met the inclusion criteria and underwent posterior spinal fixation during the study period were included, resulting in 55 patients.

Radiological evaluation

**Figure 1:**
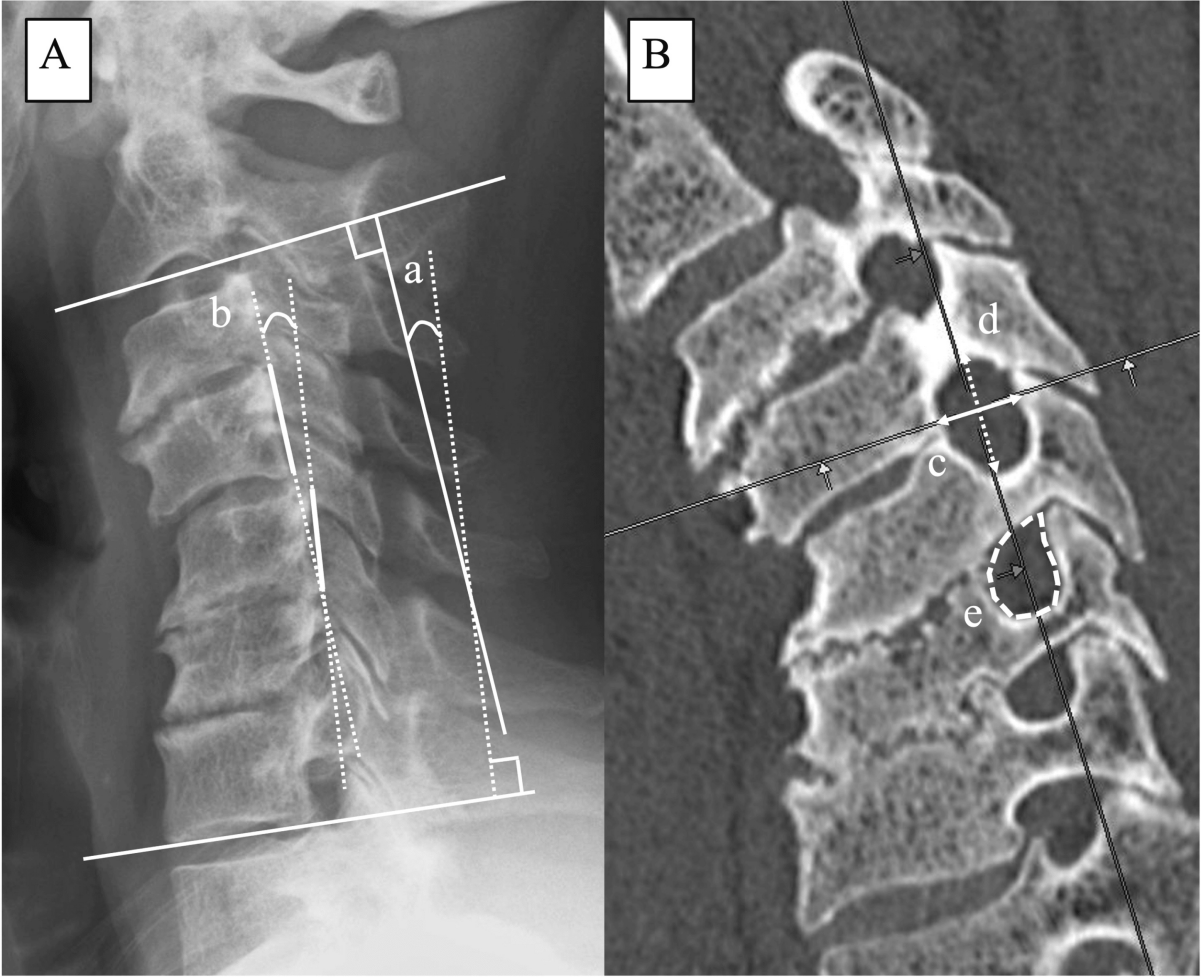
Radiological measurements and 3d-CT images. (A)  C2‒7 angle (a) and local segmental angle (b) were measured on a lateral radiograph in the neutral position. The segmental local angle (SA) was determined as the line from the posterior margins of the superior and inferior vertebrae to each targeted intervertebral foramen. (B) Sagittal CT image reconstructed using MPR.  c: The anteroposterior diameter (AP), d: craniocaudal diameter (CC), and e: foraminal area were measured using reconstructed 3D-CT images.

A total of 150 intervertebral foramina with pedicle screws inserted into both the upper and lower vertebrae were examined (foramina with screws inserted into only the upper or lower vertebrae were excluded). These included 16 at C2/3, 38 at C3/4, 48 at C4/5, 28 at C5/6, 8 at C6/7, and 12 at C7/T1 levels. Of these, 144 intervertebral foramina were measured, while six foramina were excluded due to foraminotomy at C4/5 (42 foramina at C4/5). Sagittal alignment of the cervical spine was evaluated using two parameters: C2‒7 angle and the segmental local angle (SA) on pre- and postoperative lateral radiographs in the neutral position (Figure 1A). The SA was defined by lines drawn along the posterior margins of the vertebrae superior and inferior to the targeted intervertebral foramen, with positive values indicating lordosis and negative values indicating kyphosis. The change in segmental local angle (i.e., α angle) was calculated as the difference between postoperative and preoperative SA values. Three-dimensional CT images were used to measure the anteroposterior diameter (AP, mm), craniocaudal diameter (CC, mm), and intervertebral foramen area (mm^2^) before and after surgery (Figure 1B). The CT images were obtained using the following settings: tube voltage, 120 kV; pitch factor, 0.637; and collection slice thickness, 0.5 mm. Thin-slice data were reconstructed using the following parameters: high-frequency enhancement function, FC30; reconstruction slice thickness, 1 mm; and reconstruction interval, 1 mm. The reconstructed images were uploaded to the Synapse Vincent workstation (Fujifilm Medical, Tokyo, Japan), and images perpendicular to the left and right intervertebral foramina were created using a multiplanar reconstruction (MPR).

C5 palsy

Post-operative C5 palsy was defined as a decline in deltoid and biceps muscle strength by at least one grade on manual muscle testing (MMT) without any deterioration of myelopathy. In all cases, symptoms first appeared two to three days after surgery. Patients were categorized into two groups: Group P (those who developed post-operative C5 palsy) and Group NP (those who did not).

Statistical analysis

Statistical analyses were performed using StatFlex version 6 (Artech, Osaka, Japan). Results are presented as mean ± standard deviation (SD). The C2/7 angle, SA, and diameters (CC and AP) of each foramen were compared before and after surgery using the Wilcoxon test. Statistical significance was set at p < 0.05. Spearman's correlation was used to examine changes in correlations between α angle and the percentage change in foraminal area (calculated as (postoperative area - preoperative area) / preoperative area x 100). Differences in percentage change in foraminal area across three α angle categories (α < 0°, 0° ≤ α < 10°, and α ≥ 10°) were analyzed using the Kruskal-Wallis test. Changes in CC, AP diameter, and foraminal areas were calculated by subtracting preoperative from postoperative values. Pre- and postoperative differences between groups P (C5 palsy) and NP (no C5 Palsy) were statistically examined using the Wilcoxon test.

## Results

Radiological change outcome (overall)

**Figure 2:**
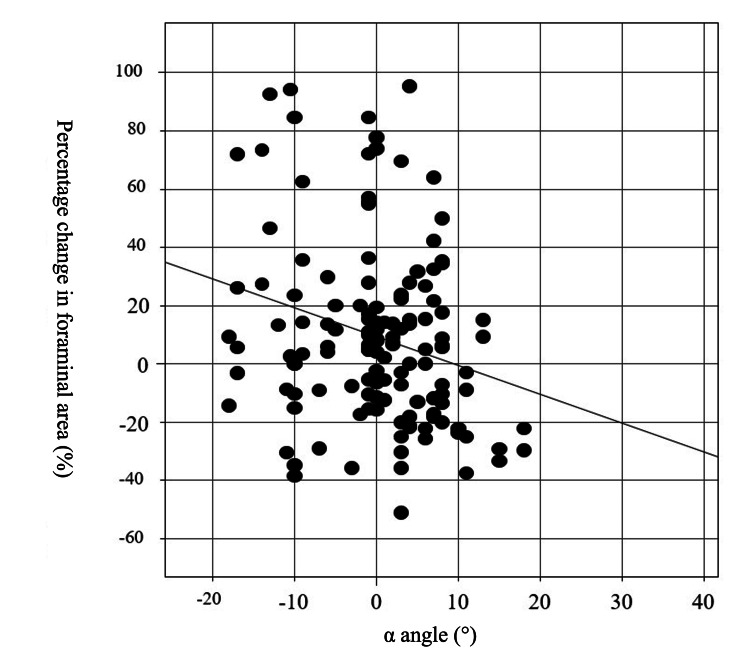
Correlation between percentage change in foraminal area and α angle of total level (C2/3 to C7/T1). Spearman rank correlation: rS = -0.2285. α angle: obtained segmental local angle postoperatively. The α angle was calculated as the postoperative segmental angle (SA) minus the preoperative SA. Large changes in segmental local angle (SA) result in narrowing of the intervertebral foraminal area.

**Figure 3:**
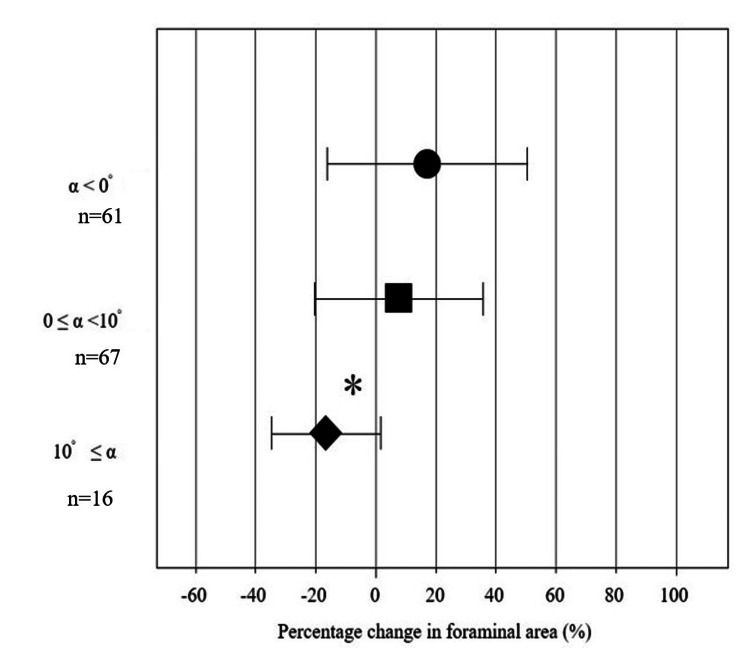
Relationship between percentage change in foraminal area (C2/3 to C7/T1) and three of α (α < 0°, 0° ≤ α < 10°, and α ≥ 10°). *: p < 0.01, Statistical significance was tested using the Kruskal–Wallis test. When the segmental angle changed by 10° or more, the intervertebral foraminal area was significantly narrower compared to the group with no change (0°).

Fifty-five patients who underwent posterior spinal fixation with screw-rod instrumentation were evaluated for 144 foramina. The mean preoperative C2-7 angle and SA were -6.2 ± 23.2° (range, 12° to -57°) and 2.3 ± 10.4° (range, 12° to -20°). In addition, the postoperative C2-7 angle changed significantly toward a lordotic alignment (Table 1, Wilcoxon test, * p = 0.017). Mean areas of the foramina were 29.2 ± 9.9 mm^2^ preoperatively and 32.3 ± 11.8 mm^2^ postoperatively. The postoperative foraminal area was significantly enlarged by an average of approximately 14.2% (3.1 mm^2^) compared to the preoperative area (Table 2, Wilcoxon test, * p = 0.0048). This change was significant. SA changed from -1.0 ± 8.7° to -0.28 ± 6.8° (NS, Table 1). Pre- and postoperative diameters were 7.8 ± 1.7 mm and 8.2 ± 1.6 mm in CC, and 3.9 ± 1.2 mm and 4.0 ± 1.6 mm in AP, respectively (Table 2). The intervertebral foraminal area expanded after cervical fixation. However, analysis of the relationship between the α angle and percentage change in foraminal area showed that the change in area tended to be smaller with a larger α angle (Figure 2; rS = -0.2285). The correlation between the percentage change in foraminal area and the α angle showed a weak negative tendency. When the α angle was less than 0°, the foraminal area was enlarged (Figure 3). The 61 patients in the 0 < group consisted of 12, 16, 19, 16, and 4 at C2/3, C3/4, C4/5, C5/6, and C7/T1, respectively. The percentage change in foraminal area (%) was compared between three categories of α angle. Intervertebral foraminal area expanded when the α angle was corrected to less than 10° or when a kyphotic alignment persisted. On the other hand, an α angle ≥ 10° resulted in significant narrowing of the foraminal area compared to an angle < 0° (non-parametric multiple group comparison, Kruskal-Wallis test, Figure 3, p < 0.05).

**Table 2:** Background Demographics of 55 patients (144 foramina) Statistical differences were examined pre- and postoperatively using the Wilcoxon test. Data were shown as Mean ± SD, C2‒7: C2‒7 angle. Pre-op: pre-operation; post-op: post-operation; post-pre: subtraction value (postoperative value minus preoperative value); Area: foraminal area; SA: segmental local angle; %area: percentage change in foraminal area; AP: Anteroposterior diameter; CC: Craniocaudal diameter. post-pre: Amount of change pre- and postoperatively. Measurements of 144 foramen samples from 55 patients are presented.

	pre-op	post-op	post-pre	p
C2‒7 (°)	-6.2± 23.2	2.3 ± 10.4	8.2 ± 21.9	0.017
Area (mm^2^)	29.2 ± 9.9	32.3 ± 11.8	3.1 ± 8.4	0.0048
%area (%)	-	-	14.2 ± 31.2	
SA (°)	-1.0± 8.7	-0.28 ± 6.8	0.7 ± 8.4	NS
AP (mm)	3.9 ± 1.2	4.0 ± 1.6	0.05 ± 1.2	NS
CC (mm)	7.8 ± 1.7	8.2 ± 1.6	0.42 ± 1.4	NS

Radiological change outcome at C4/5

Mean preoperative SA were -6.0 ± 8.9° (range, 11° to -26°) and 1.0 ± 7.0° (range, 16° to -8°). Postoperative SA changed significantly to a lordotic angle (Table 3, Wilcoxon test, * p < 0.05). Mean areas of the foramina were 30.5 ± 9.1 mm^2^ preoperatively and 29.6 ± 9.5 mm² postoperatively. The postoperative foraminal area was smaller by an average of about -2.9% (-0.8 mm^2^) compared to the preoperative area (Table 3, NS). Pre- and postoperative diameters were 8.1 ± 1.7 mm and 8.2 ± 1.4 mm in CC, and 3.8 ± 1.1 mm and 3.6 ± 1.5 mm in AP, respectively (Table 3). Postoperative AP diameter was slightly shorter than before surgery. 

**Table 3:** Demographic data of 55 patients, 144 foramina Results of 144 foraminal measurements are presented. Statistical differences pre- and postoperatively were examined using the Wilcoxon test. * p<0.05. Data were shown as Mean ± SD, C2‒7: C2‒7 angle. Pre-op: pre-operation; post-pre: subtraction value (postoperative value minus preoperative value); Area: foraminal area; SA: segmental local angle; %area: percentage change in the foraminal area; AP: Anteroposterior diameter; CC: Craniocaudal diameter; post-pre: Amount of change pre- and postoperatively. Post-pre of SA, denoted as α angle: subtraction value postoperative SA minus preoperative SA.

Level		SA (°)	Area (mm^2^)	%area (%)	AP (mm)	CC (mm)
C2/3 n = 16	pre-op	-0.9 ± 8.0	34.6 ± 10.8	-	4.6 ± 1.3	8.6 ± 1.9
	post-op	4.3 ± 5.2	40.8 ± 11.3	-	4.8 ± 1.7	9.3 ± 1.4
	post-pre	3.4 ± 5.6	6.2 ± 6.5	22.0 ± 29.3	0.2 ± 0.9	0.7 ± 0.8
	p	NS	0.001		NS	NS
C3/4 n = 38	pre-op	1.8 ± 10.1	26.1 ± 7.6	-	3.3 ± 0.9	7.6 ± 1.5
	post-op	0.1 ± 7.3	27.6 ±9.8	-	3.4 ± 1.2	7.9 ± 1.5
	post-pre	-1.7 ± 8.7	1.5 ± 8.1	8.0 ± 31.1	0.05 ± 0.9	0.3 ± 1.4
	p	NS	NS		NS	NS
C4/5 n = 42	pre-op	-6.0 ± 8.9	30.5 ± 9.1	-	3.8 ± 1.1	8.1 ± 1.7
	post-op	1.0 ± 7.0	29.6 ± 9.5	-	3.6 ± 1.5	8.2 ± 1.4
	post-pre	7.0 ± 9.8 *	-0.8 ± 8.0	-2.9± 8.0	-0.3 ± 1.1	0.08 ± 1.2
	p	0.005	NS	-	NS	NS
C5/6 n = 28	pre-op	-3.0 ± 6.6	27.0 ± 10.7	-	3.5 ± 1.0	7.7 ± 1.4
	post-op	-4.0 ± 6.3	33.4 ± 10.2	-	4.0 ± 1.9	8.4 ± 1.5
	post-pre	-1.0 ± 6.3	6.4 ± 7.4	31.1 ± 34.2	0.5 ± 1.5	0.7 ± 1.6
	p	NS	0.001		NS	NS
C6/7 n = 8	pre-op	5.8 ± 6.8	32.6 ± 7.2	-	4.1 ± 1.1	8.5 ± 1.0
	post-op	3.2 ± 8.2	39.0 ± 14.2	-	4.6 ± 1.5	9.1 ± 1.6
	post-pre	-2.6 ± 13.9	6.4 ± 10.5	19.2 ± 33.5	0.4 ± 1.2	0.6 ± 1.4
	p	NS	NS		NS	NS
C7/T1 n = 12	pre-op	-0.2 ± 7.3	28.5 ± 13.7	-	4.9 ± 1.0	6.4 ± 2.1
	post-op	-0.5 ± 4.6	31.5 ± 12.0	-	4.5 ± 1.3	6.5 ± 1.5
	post-pre	-0.3 ± 5.2	3.0 ± 8.9	14.6 ± 25.8	-0.4 ± 1.3	0.1 ± 2.1
	p	NS	NS		NS	NS

C5 palsy

Table 4 summarizes radiological findings in patients with C5 palsy. In the C5 paralysis group, no new signal intensity changes were observed in the spinal cord on postoperative T2-weighted MRI. Five of the seven patients fully recovered to MMT grade 5, one recovered to grade 4, and 1 to grade 3 within one year post-surgery. Age, sex, and pre- and post-pre CC diameters showed no significant differences between Groups P and NP (Table 4). However, post-pre SA (α angle) of Group P (9.5 ± 10.9°) was significantly bigger than in Group NP (3.1 ± 11.4°). In addition, the decrease in foraminal area and AP diameter in Group P (-8.6 ± 7.5 mm^2^, and -1.2 ± 1.1 mm, respectively) was significantly greater than in Group NP (0.66 ± 7.3 mm^2^, and -0.07 ± 1.3 mm, respectively). The area in Group P was approximately 26.9% (8.6 mm^2^) smaller than the preoperative area (p < 0.05).

**Table 4:** Demographic data of C5 P group and NP group 42 foramia at C4/5 Data were shown as Mean ± SD, (95% CIs), C2‒7: C2‒7 angle. The spinal canal area became narrowed after surgery in P (+) group. Pre- and postoperative differences between groups P and NP were statistically examined using the Wilcoxon test. * p < 0.05. Pre-op: pre-operation, post-op: post-operation, post-pre: subtraction value, i.e., postoperative value minus preoperative value; Area: foraminal area, SA: segmental local angle, %area: percentage change of foraminal area, AP: Anteroposterior diameter, CC: Craniocaudal diameter. Pre-post-op: Amount of change in pre- and post-operation.

	age		SA ( °)	Area (mm^2^)	%area (%)	AP (mm)	CC (mm)
NP n = 35	69.1	pre-op	-4.3 ± 9.7 (-8.9 - 0.3)	30.2 ± 9.4 (36.6 – 23.7)	-	3.9 ± 1.1 (4.2 – 3.5)	8.0 ± 1.8 (8.5 – 7.4)
		post-op	-1.1 ± 6.8 (-4.3 – 2.1)	30.9 ± 9.7 (27.7 – 33.9)	-	3.8 ± 1.5 (3.3 – 4.3)	8.3 ± 1.7 (7.8 – 8.8)
		Post-pre	3.1 ± 11.4 (-2.3 – 8.5)	0.66 ± 7.3 (-1.6 - 3.0)	5.3 ± 27.4	-0.07 ± 1.3 ( -0.3 – 0.26)	0.2 ± 1.3 (-0.15 – 0.6)
		p	NS	NS	-	NS	NS
P n = 7	71.0	pre-op	-6.1± 11.0 (-13.3 – 1.2)	31.9± 7.7 (26.5 – 37.1)	-	3.6± 1.0 (4.3 – 2.9)	8.5 ± 1.2 (7.7 – 9.3)
		post-op	3.5 ± 6.6 (-0.9 – 7.8)	23.2± 5.9 (19.2 – 27.2)	-	2.5 ± 0.9 (1.8 - 3.0)	7.8 ± 0.9 (7.1 - 8.4)
		post-pre	9.5 ± 10.9 * (2.3 – 16.7)	-8.6 ± 7.5 * (-13.7 - -3.4)	-26.9 ± 17.4*	-1.2 ± 1.1 * (-1.9 - -0.3)	-0.7 ± 1.0 (-1.5 - -0.04)
		p	0.019	0.031	-	0.046	NS

## Discussion

C5 nerve root palsy is a common complication of cervical surgery using both anterior and posterior approaches. It is frequently observed after posterior fixation or corrective surgery [6-8,19,20]. Several factors may contribute to C5 palsy, and previous reports have suggested five pathological mechanisms: 1) nerve root traction caused by spinal cord shifting following wide laminectomy [8,11-12]; 2) spinal cord ischemia due to reduced blood supply from radicular arteries [20]; 3) excessive kyphotic correction [4,5]; 4) segmental spinal cord disorder [9,21]; and 5) spinal cord reperfusion injury [22,23]. Each of these has been proposed as a potential cause of C5 palsy in prior reviews. Whether occurring simultaneously or independently, these mechanisms occur with high frequency after surgery, such as CSM with fixation surgery (12%) and LP for ossification of the posterior longitudinal ligament (OPLL, 21.4%). The incidence of C5 palsy among patients with CSM differed between the LP (4.4%) and LP combined with fusion (LPF) (12.2%) [13,14]. These patients included those with OPLL and CSM who underwent either anterior or posterior surgery. To better isolate contributing factors, we believe that pure postoperative C5 palsy should be examined in a more specific population. Therefore, we conducted a study limited to C5 palsy occurring after cervical fixation using CPSs in patients with CSM. Although cervical correction surgery is known to narrow the foraminal area, the mechanism and extent of this narrowing remain unclear. To clarify this issue, we evaluated the foraminal area as a percentage change from the preoperative area. Percentage change was used because some asymptomatic patients already had narrow foramina preoperatively. In the present study, the foraminal area was measured using 3DCT by two specialists. This modality allows for accurate depiction of the intervertebral foramina by correcting the inclination angle along the endplate of the cervical spine in both sagittal and coronal planes. Accurate axial CT images cannot be obtained using 2DCT, whereas 3DCT can more precisely evaluate the foraminal area, particularly in patients with kyphotic alignment. Although the postoperative intervertebral foraminal area was significantly enlarged as a whole, a negative correlation was observed between α angle and postoperative foraminal area (Figure 2). One key finding was that the foraminal area was significantly smaller when the α angle was ≥ 10°, with a mean reduction in postoperative area of 16.4% (5.4 mm^2^) compared to the preoperative area (Figure 3). This phenomenon was also observed in C4/5, where the intervertebral foraminal area decreased as the α angle was about 10 degrees in Group P. The post-pre change in area and AP diameter in Group P (-8.6 ± 7.5 mm^2^, and -1.2 ± 1.1 mm, respectively) were significantly smaller than in Group NP, and the percent area decreased by 26.9% (8.6 mm^2^) (Table 4). When the α angle approached 10 degrees, foraminal narrowing became significant. We initially hypothesized that craniocaudal narrowing occurs after correction surgery using compression and the cantilever technique with a CPS. However, the AP diameter of the foramen tended to shorten after surgery (p < 0.05), whereas the CC diameter showed no change. This was confirmed by 3DCT, which showed that the mechanism of foraminal stenosis involves a “lag effect” where the fixed upper vertebra is pulled backward, causing posterior slippage and leading to AP foraminal stenosis. In such cases, foraminotomy that decompresses the AP diameter at the foramen could be necessary [16,20,24]. Surgical correction of kyphosis offers good efficacy when the correction targets the apex of kyphosis; however, the risks increase with the magnitude of correction. This study included patients who underwent posterior fixation using pedicle screws. However, A limitation of this study was that the investigated individuals comprised those who underwent corrective surgery and those who underwent short intervertebral fixation (with instability). The present study also demonstrates that major changes in the correction angle of most kyphotic segments should be avoided during kyphosis correction or fixation surgery. In other words, the division of the target correction angle into several segments is required to obtain a large C2-7 angle.

## Conclusions

This study contributes to a better understanding of the impacts of changes in the foraminal area after posterior fusion using CPS and shows a potential pathway to reduce the risk of nerve root palsy caused by narrowing of the foraminal areas.
